# Neurophysiological profiles of patients with bipolar disorders as probed with transcranial magnetic stimulation: A systematic review

**DOI:** 10.1002/npr2.12458

**Published:** 2024-06-26

**Authors:** Keita Taniguchi, Naotsugu Kaneko, Masataka Wada, Sotaro Moriyama, Shinichiro Nakajima, Masaru Mimura, Yoshihiro Noda

**Affiliations:** ^1^ Department of Neuropsychiatry Keio University School of Medicine Tokyo Japan; ^2^ Department of Life Sciences, Graduate School of Arts and Sciences The University of Tokyo Tokyo Japan

**Keywords:** bipolar disorders, depression, electroencephalography, neurophysiology, stimulation methods, transcranial magnetic stimulation

## Abstract

**Aim:**

Bipolar disorder (BD) has a significant impact on global health, yet its neurophysiological basis remains poorly understood. Conventional treatments have limitations, highlighting the need for a better understanding of the neurophysiology of BD for early diagnosis and novel therapeutic strategies.

**Design:**

Employing a systematic review approach of the PRISMA guidelines, this study assessed the usefulness and validity of transcranial magnetic stimulation (TMS) neurophysiology in patients with BD.

**Methods:**

Databases searched included PubMed, MEDLINE, Embase, and PsycINFO, covering studies from January 1985 to January 2024.

**Results:**

Out of 6597 articles screened, nine studies met the inclusion criteria, providing neurophysiological insights into the pathophysiological basis of BD using TMS–electromyography and TMS–electroencephalography methods. Findings revealed significant neurophysiological impairments in patients with BD compared to healthy controls, specifically in cortical inhibition and excitability. In particular, short‐interval cortical inhibition (SICI) was consistently diminished in BD across the studies, which suggests a fundamental impairment of cortical inhibitory function in BD. This systematic review corroborates the potential utility of TMS neurophysiology in elucidating the pathophysiological basis of BD. Specifically, the reduced cortical inhibition in the SICI paradigm observed in patients with BD suggests gamma‐aminobutyric acid (GABA)‐A receptor‐mediated dysfunction, but results from other TMS paradigms have been inconsistent. Thus, complex neurophysiological processes may be involved in the pathological basis underlying BD. This study demonstrated that BD has a neural basis involving impaired GABAergic function, and it is highly expected that further research on TMS neurophysiology will further elucidate the pathophysiological basis of BD.

## Abstract 2

This systematic review examined the neurophysiological profiles of patients with bipolar disorder (BD) using transcranial magnetic stimulation. The review found that patients with BD had consistently reduced short‐interval cortical inhibition compared to healthy controls (HCs), suggesting gamma‐aminobutyric acid (GABA)‐A receptor‐mediated neurophysiological function may be impaired. In addition, decreased long‐interval intracortical inhibition and cortical silent period were observed in patients with BD compared to HC, suggesting GABA‐B receptor‐mediated neurophysiological impairment. These results indicate that impaired GABAergic neurophysiological function may underlie the important pathophysiological basis of the disorder in patients with BD.

## Graphical abstract

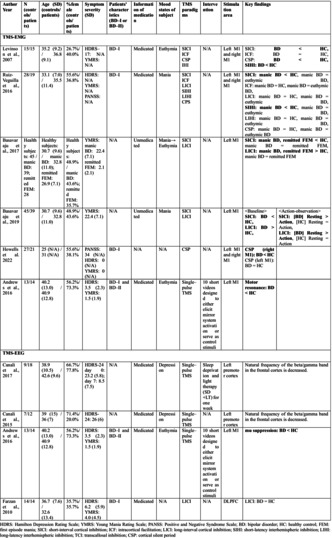

## 1 INTRODUCTION

Bipolar disorder (BD) is a psychiatric disorder that affects nearly 1% of the global population.^1^ Epidemiological studies report that life expectancy may be 10 to 20 years shorter in patients with BD, as well as in patients with schizophrenia.^2^, ^3^ Furthermore, while BD is one of the major psychiatric disorders, most patients with BD are not accurately diagnosed until approximately 6 to 10 years after their first visit to a healthcare provider.^4^ In addition, lithium and other drug therapies have been the mainstay of treatment for BD but are suboptimal due to the presence of treatment‐resistant cases and problems with side effects. Therefore, there is an urgent need to elucidate the neurophysiological basis of BD to enable early diagnosis of BD and the development of novel therapies.

Research into the pathophysiology of BD involves a wide range of research methods, each providing unique insights. Magnetic resonance imaging (MRI) studies have reported widespread cortical thinning, highlighting structural abnormalities in BD.^5^ Additionally, functional MRI (fMRI) investigations reveal altered activity in key brain areas, for example, variable amygdala activation depending on mood states and consistent hypoactivity in the prefrontal cortex.^6^ These changes are linked to different emotional conditions. Moreover, PET studies show increased binding in the right hippocampus of BD type I patients, suggesting specific regional disruptions.^7^ Magnetic resonance spectroscopy (^1^H‐MRS) and genetic studies further our understanding by showing alterations in brain metabolites and shared genetic variants,^8^ notably in calcium channel signaling genes.^9^ Additionally, research into cerebrospinal fluid and plasma in pediatric BD patients connects neuroinflammatory markers and oxidative stress to cognitive impairments in mood regulation.^10^ Finally, studies on auditory steady‐state responses (ASSRs) indicate reduced neural synchrony in BD, particularly at key gamma frequencies.^11^, ^12^ Together, these studies contribute to a comprehensive view of BD, increasing our knowledge of both central and peripheral changes and paving the way for advances in diagnostics and treatments.

Traditional approaches in studying BD have primarily focused on static measurements of brain structure and neurotransmitter levels. While fMRI and EEG provide insights into the dynamic functions of the brain, they fall short of elucidating the causal physiological roles associated with neurotransmitters. Transcranial magnetic stimulation (TMS) combined with EEG (TMS‐EEG) and EMG (TMS‐EMG) overcomes these limitations by providing a direct assessment of neurotransmitter‐specific activities and dynamic neural processes. In fact, the TMS neurophysiology has been applied to a variety of psychiatric disorders, and disease‐specific neural activity has gradually become apparent.^13^, ^14^, ^15^, ^16^, ^17^, ^18^, ^19^ This innovative approach improves our understanding of the pathophysiology of BD beyond conventional neuroimaging and biochemical techniques, thus enriching psychiatric research.

These methods enhance our understanding of the neurophysiology of BD, specifically highlighting real‐time neural activity and neurotransmitter‐specific functions related to glutamatergic and gamma‐aminobutyric acid (GABA)ergic systems. Moreover, TMS‐EEG and TMS‐EMG can effectively probe cortical excitability and inhibition (E/I), providing critical insights into the neurophysiological E/I balance in BD.^15^, ^20^ Furthermore, the versatility of TMS paradigms allows for detailed investigation of cortical function and neuroplasticity. Several established TMS paradigms enable us to investigate the specific neurotransmitter‐related receptor functions by varying stimulus parameters, including conditioned stimulus (CS) and test stimulus (TS) intensities, as well as interstimulus intervals (ISIs). Table 1 shows the typical TMS neurophysiological paradigms. TMS neurophysiology also could overcome the limitations of traditional neuroimaging and biochemical analyses by indexing the neurotransmitter systems beyond glutamatergic and GABAergic functions, such as cholinergic function and neuroplasticity in targeted brain regions. Therefore, the TMS‐EEG and TMS‐EMG methods provide a more comprehensive neuroscientific tool for assessing various neurophysiological functions in BD.^21^, ^22^, ^23^

**TABLE 1:** Summary of the TMS paradigms.

Paired‐pulse					
TMS paradigm	SICI	LICI	SIHI	LIHI	ICF
CS intensity	Sub‐threshold TMS	Supra‐threshold TMS	Supra‐threshold TMS–contra‐hemisphere	Supra‐threshold TMS–contra‐hemisphere	Sub‐threshold TMS
TS intensity	Supra‐threshold TMS	Supra‐threshold TMS	Supra‐threshold TMS	Supra‐threshold TMS	Supra‐threshold TMS
ISI (ms)	2–5	50–200	8–20	40–50	8–30
Proposed neurotransmitter/receptor/neural changes	GABA‐A	GABA‐B	Not known	GABA‐B	Glu

Single‐pulse					
TMS paradigm	CSP	ISP	SAI	LAI	PAS
Stimulation intensity	Supra‐threshold	Supra‐threshold	Supra‐threshold	Supra‐threshold	Supra‐threshold
Subject condition	Voluntary contraction of the FDI muscle contralateral to the stimulus at a constant strength	Voluntary contraction of the FDI muscle ipsilateral to the stimulus at a constant strength	Electrical stimulation of the contralateral peripheral nerve was performed approximately 20 ms before TMS	Electrical stimulation of the contralateral peripheral nerve was performed approximately 200 ms before TMS	Electrical stimulation of the contralateral peripheral nerve was performed 10–25 ms before TMS
Proposed neurotransmitter/receptor/neural changes	GABA‐B	GABA‐B	GABA‐A ACh	Not known	LTP/LTD

Abbreviations: Ach, acetylcholine; CS, condition stimulus; CSP, cortical silent period; GABA, γ‐aminobutyric acid; Glu, glutamine; ICF, intracortical facilitation; ISI, interstimulus interval; ISP, ipsilateral silent period; LAI, long‐latency afferent inhibition; LICI, long‐interval cortical inhibition; LIHI, long‐latency interhemispheric inhibition; LTD, long‐term depression; LTP, long‐term potentiation; PAS, paired associative stimulation; SAI, short‐latency afferent inhibition; SICI, short‐interval cortical inhibition; SIHI, short‐latency interhemispheric inhibition; TCI, transcallosal inhibition; TS, test stimulus.

The number of TMS neurophysiological studies in various psychiatric disorders has increased significantly in recent years. However, there has not yet been a comprehensive review of the evidence in relation to BD. The aim of this systematic review was to qualitatively assess existing TMS studies. We will focus on assessing the robustness of experimental designs comparing patients with BD with HC and critically analyze the methodologies, particularly the TMS parameters employed. This evaluation will not only provide a clearer understanding of the neurophysiological aspects of BD but also contribute to identifying methodological consistencies and discrepancies. Furthermore, it will highlight potential directions for future TMS research in BD, underscoring areas that require further investigation and clarifying how these studies contribute to our overall understanding of the disorder. Furthermore, the intent of this study was to delineate the significance of this specialty by providing a comprehensive landscape of TMS research in BD.

## 2 METHODS

### 2.1 Search strategy

This systematic review was conducted following the Preferred Reporting Items for Systematic Reviews and Meta‐Analyses (PRISMA) guidelines.^24^ We searched using PubMed, MEDLINE, Embase, and PsycINFO, covering the period from January 1985 to January 2024, and filtered to exclude research articles written in English and involving human subjects. The search terms included (TMS OR (transcranial magnetic stimulation)) AND ((brain activity) OR (brain waves) OR (EEG OR electroencephalog*)) OR ((EMG OR electromyog*) OR (MEP OR (motor evoked potential)) OR (TMS‐EEG) OR (TMS‐EMG) OR (neurophysiol*) OR (neuroplasticity OR plasticity OR plastic) OR ((short interval intracortical inhibition) OR SICI) OR ((intracortical facilitation) OR ICF) OR ((long interval intracortical inhibition) OR LICI) OR ((paired associative stimulation) OR PAS) OR ((short latency afferent inhibition) OR SAI) OR ((cortical silent period) OR CSP) OR ((ipsilateral silent period) OR iSP) OR ((interhemispheric inhibition) OR IHI)) AND ((bipolar disorder) OR (bipolar depression) OR (bipolar affective disorder) OR (manic depressive impairment)).

### 2.2 Inclusion criteria

We included the studies in our systematic review if they adhered to the following criteria: (1) Patients with BD were diagnosed by operational diagnostic criteria; (2) TMS‐EEG or TMS‐EMG was conducted using any of the following paradigms: single pulse, SICI, LICI, ICF, CSP, ISP, IHI, SAI, LAI, and PAS; and (3) results were included for both patients with BD and HC. On the other hand, studies exclusively utilizing TMS for motor threshold (MT) measurement were excluded in this study, as meta‐analyses for MT in patients with BD have been previously reported.^25^ The data extraction process involved two stages: K.T. conducted the first screening, and S.M. performed the second screening. In cases where discrepancies arose between the two screenings, they were reviewed and resolved by our senior researcher, Y.N.

### 2.3 Data extraction

In this review, we extracted data for each study on the author's name, year of publication, number of subjects, mean age, percentage of females, severity of depression, patients' characteristics, TMS paradigms, analytical method, intervention, stimulation area, and key findings. These were further classified into (1) TMS‐EMG and (2) TMS‐EEG and summarized in Table 2.

**TABLE 2:** Characteristics of included studies.

Author, year	*N* (controls/patients)	Age (SD) (controls/patients)	%female (controls/patients)	Symptom severity (SD)	Patients' characteristics (BD‐I or BD‐II)	Information of medication	Mood states of subject	TMS paradigms	Intervention	Stimulation area	Key findings	
TMS‐EMG												
Levinson et al. (2007)	15/15	35.2 (9.2)/36.8 (9.1)	26.7%/40.0%	HDRS: 17: N/A YMRS: N/A	BD‐I	Medicated	Euthymia	SICI ICF CSP IHI	N/A	Left M1 and right M1	SICI: **BD < HC,** ICF: BD = HC, CSP: **BD < HC,** **SIHI: BD < HC**	
Ruiz‐Veguilla et al. (2016)	28/19	33.1 (7.0)/35.5 (11.4)	55.6%/36.8%	HDRS: N/A YMRS: N/A PANSS: N/A	BD‐I	Medicated	Mania	SICI ICF LICI SIHI LIHI CPS	N/A	Left M1 and right M1	**SICI: manic BD < HC**, manic BD = euthymic BD, ICF: manic BD = HC, manic BD = euthymic BD, LICI: manic BD = HC, manic BD = euthymic BD, **SIHI: manic BD < HC**, manic BD = euthymic BD, LIHI: manic BD = HC, manic BD = euthymic BD, CSP: manic BD = HC, manic BD = euthymic BD	
Basavaraju et al. (2017)	Healthy subjects: 45/manic BD: 39; remitted FEM: 28	Healthy subjects: 30.7 (9.6)/manic BD: 32.8 (11.0); remitted FEM: 26.9 (7.1)	Healthy subjects: 48.9%/manic BD: 43.6%; remitted FEM: 35.7%	YMRS: Manic BD: 22.4 (7.1) Remitted FEM: 2.1 (2.1)	N/A	Unmedicated	Mania → Euthymia	SICI LICI	N/A	Left M1	**SICI: manic BD, remitted FEM < HC**, manic BD = remitted FEM, **LICI: manic BD, remitted FEM > HC**, manic BD = remitted FEM	
Basavaraju et al. (2019)	45/39	30.7 (9.6)/32.8 (11.0)	48.9%/43.6%	YMRS: 22.4 (7.1)	N/A	Unmedicated	Mania	SICI LICI	N/A	Left M1	<Baseline> **SICI: BD < HC, LICI: BD > HC**	<Action observation> **SICI: [BD] Resting > Action**, [HC] Resting = Action, **LICI: [BD] Resting > Action**, [HC] Resting = Action
Howells et al. (2022)	27/21	25 (N/A)/31 (N/A)	55.6%/38.1%	PANSS: 34 (N/A) HDRS: 0 (N/A) YMRS: 0 (N/A)	BD‐I	N/A	N/A	CSP	N/A	Left M1 and right M1	**CSP (right M1): BD < HC** CSP (left M1): BD = HC	
Andrews et al. (2016)	13/14	40.2 (13.0)/40.9 (12.8)	56.2%/73.3%	HDRS: 3.5 (2.3) YMRS: 1.5 (1.9)	BD‐I and BD‐II	Medicated	Euthymia	Single‐pulse TMS	10 short videos designed to either elicit mirror system activation or serve as control stimuli	Left M1	**Motor resonance: BD < HC**	
TMS‐EEG												
Canali et al. (2017)	9/18	38.9 (10.5)/42.6 (9.6)	66.7%/77.8%	HDRS: 24 day 0: 23.2 (5.8); day 7: 8.5 (7.5)	N/A	Medicated	Depression	Single‐pulse TMS	Sleep deprivation and light therapy (SD + LT) for 1 week	Left premotor cortex	Natural frequency of the beta/gamma band in the frontal cortex is decreased	
Canali et al. (2015)	7/12	39 (15)/36 (7)	71.4%/20.0%	HDRS: 24: 26 (6)	N/A	Medicated	Depression	Single‐pulse TMS	N/A	Left premotor cortex	Natural frequency of the beta/gamma band in the frontal cortex is decreased	
Andrews et al. (2016)	13/14	40.2 (13.0)/40.9 (12.8)	56.2%/73.3%	HDRS: 3.5 (2.3) YMRS: 1.5 (1.9)	BD‐I and BD‐II	Medicated	Euthymia	Single‐pulse TMS	10 short videos designed to either elicit mirror system activation or serve as control stimuli	Left M1	**mu suppression: BD < HC**	
Farzan et al. (2010)	14/14	36.7 (7.6)/32.6 (13.4)	35.7%/35.7%	HDRS: 6.2 (5.9) YMRS: 4.0 (4.5)	BD‐I	Medicated	N/A	LICI	N/A	DLPFC	LICI: BD = HC	

Abbreviations: BD, bipolar disorder; CSP, cortical silent period; FEM, first‐episode mania; HC, healthy control; HDRS, Hamilton Depression Rating Scale; ICF, intracortical facilitation; LICI, long‐interval cortical inhibition; LIHI, long‐latency interhemispheric inhibition; PANSS, Positive and Negative Syndrome Scale; SICI, short‐interval cortical inhibition; SIHI, short‐latency interhemispheric inhibition; TCI, transcallosal inhibition; YMRS, Young Mania Rating Scale.

### 2.4 Risk of bias

We conducted the risk of bias assessment using the specific tool for non‐randomized studies^26^ for the following factors: (1) participant selection, (2) confounding variables, (3) measurement of exposure, (4) blinding of outcome assessments, (5) incomplete outcome data, and (6) selective outcome reporting.

## 3 RESULTS

**FIGURE 1:**
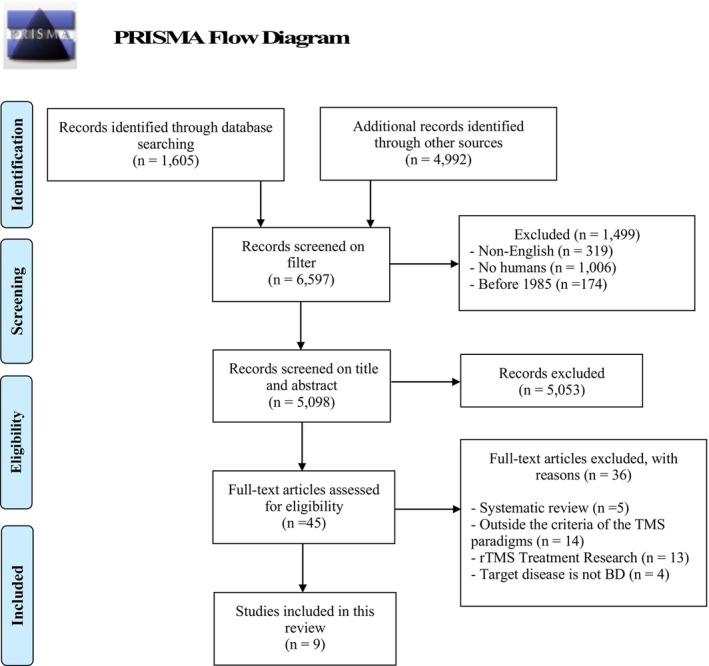
PRISMA flowchart for this systematic review. This diagram shows a flowchart illustrating the process of identifying the final extracted articles in this study.

The initial search identified 6597 articles, and nine studies were finally included in this study. Among them, six studies examined TMS neurophysiology using TMS‐EMG^27^, ^28^, ^29^, ^30^, ^31^, ^32^ and four studies examined TMS neurophysiology using TMS‐EEG^27^, ^29^, ^33^, ^34^ for patients with BD. Of note, one study examined TMS neurophysiology using both TMS‐EMG and TMS‐EEG methods.^27^ The PRISMA flow diagram for this systematic review and meta‐analysis is shown in Figure 1. Regarding the risk of bias assessment, the risk was “low” in all four categories, except “confounding variables,” which was judged to be high risk because the effect of medication could not be ruled out in the seven studies,^27^, ^29^, ^30^, ^31^, ^32^, ^33^, ^34^ and “selective outcome reporting,” which was judged to be unknown because the experimental protocol was not available (see Figure S1).

### 3.1 TMS‐EMG studies

#### 3.1.1 Breakdown of TMS‐EMG paradigms

Among the studies included in this review, six studies investigated TMS neurophysiology with TMS‐EMG.^27^, ^28^, ^30^, ^31^, ^32^, ^35^ Specifically, SICI was investigated in four studies,^28^, ^31^, ^32^, ^35^ LICI in three studies,^28^, ^29^, ^32^, ^35^ ICF in two studies,^31^, ^32^ and CSP in three studies.^30^, ^31^, ^32^ In addition, two studies examined transcallosal inhibition (TCI) using the interhemispheric inhibition (IHI) paradigm,^31^, ^32^ but none of the studies examined ISP. There were no studies that compared the BD and HC groups using the short‐latency afferent inhibition (SAI), long‐latency afferent inhibition (LAI), and paired associative stimulation (PAS) paradigms. One study used motor evoked potentials (MEPs) to assess interpersonal motor resonance ability, which is associated with the mirror neuron system.^27^

#### 3.1.2 The area of brain stimulation and stimulation intensity

Of the TMS‐EMG studies included in this review, in the SICI and ICF paradigms, one study set the intensity of the CS at 80% of resting motor threshold (RMT),^31^ while one study set it at 80% of active motor threshold.^32^ TS was set to stimulation intensity to elicit a 1 mV peak‐to‐peak MEP amplitude in all studies that performed SICI and ICF paradigms.^28^, ^31^, ^32^, ^35^ In the LICI and IHI paradigms, both CS intensities were set to 1 mV peak‐to‐peak MEP amplitude. On the other hand, for TS in the LICI and IHI paradigms, the intensity that elicits 1 mV peak‐to‐peak MEP amplitude or 120% RMT was used.^28^, ^31^, ^32^, ^35^ In the CSP paradigm, one study stimulated M1 with titrating stimulus intensities of 110%, 120%, 130%, and 140%^31^ RMT while the first dorsal interosseous muscle was continuously contracted at 20% of maximal voluntary contraction. On the other hand, one study stimulated M1 with 110%, 120%, and 140% RMT, randomizing strength, and hemisphere, while continuously contracting the abductor pollicis brevis muscle at 40%.^30^ Another study stimulated M1 with stimulus intensity set at 120% RMT.^32^ In addition, one study that examined interpersonal motor resonance ability used the stimulus intensity of 120% RMT to measure MEP.^27^ To determine stimulus intensity, twitch of the right first dorsal interosseous muscle was used as an indicator in five studies,^27^, ^28^, ^31^, ^32^, ^35^ while in the remaining one study the abductor pollicis brevis muscle was used as an indicator.^30^ In the IHI paradigm, the right M1 was also stimulated because the IHI paradigm requires bilateral brain stimulation.^31^, ^32^

#### 3.1.3 The results of the SICI paradigm

All four studies^28^, ^31^, ^32^, ^35^ that examined the SICI paradigm showed decreased cortical inhibition in the BD group compared with the HC group, regardless of the mood phase (manic or euthymic phase) or clinical subtypes (BD type I or BD type II). Of note, none of the studies included patients with BD in the depressed phase.

#### 3.1.4 The results of the ICF paradigm

Neither of the two studies^31^, ^32^ that examined the ICF paradigm showed any significant differences between the BD and HC groups.

#### 3.1.5 The results of the LICI paradigm

One study reported no significant differences between the BD and HC groups,^32^ whereas the other two studies^28^, ^35^ showed greater cortical inhibition in the BD group, including the manic and euthymic phases, compared with the HC group.

#### 3.1.6 The results of the CSP paradigm

Three studies examined the CSP paradigm for the BD group. Levinson et al.^31^ reported a shortening of CSP in patients with BD in the euthymic phase compared with HCs, whereas Ruiz‐Veguilla et al.^32^ found no significant difference between patients with BD in the manic phase and HCs. Howells et al.^30^ included patients with BD‐I who had a history of psychosis and reported a significant shortening of CSP when the right M1 was stimulated; however, there was no significant difference in CSP between BD‐I and HCs when the left M1 was stimulated.

#### 3.1.7 The results of the IHI paradigm

Two studies that examined the short‐latency interhemispheric inhibition (SIHI) in patients with BD type I showed significant reductions in TCI.^31^, ^32^ However, these two studies targeted different phases of BD. Levinson et al. examined euthymic BD, and Ruiz‐Veguilla et al. examined manic BD. On the other hand, no significant group differences were found for the long‐latency interhemispheric inhibition (LIHI) paradigm in either study.

#### 3.1.8 The result of the interpersonal motor resonance ability as indexed by single‐pulse TMS paradigm

There was one study that investigated interpersonal motor resonance and the mirror neuron system.^27^ In this study, specific M1 areas were stimulated using TMS while participants watched others in action, and their responses of brain activity to them were recorded using EEG. Significantly less mu suppression was observed in the BD group compared to the HC group, but there were no significant differences between the two groups in terms of motor resonance.

### 3.2 TMS‐EEG studies

#### 3.2.1 Breakdown of TMS‐EEG paradigms

Three of the four studies examined single‐pulse TMS,^27^, ^33^, ^34^ and only one study investigated the LICI paradigm.^29^

#### 3.2.2 The area of brain stimulation and stimulation intensity

All four studies targeted the left hemisphere.^27^, ^29^, ^33^, ^34^ Two studies targeted the left premotor cortex,^33^, ^34^ while the other two studies targeted the left M1.^27^, ^29^ In addition, one study performed TMS‐EEG targeting the left DLPFC.^29^ Two studies used the Navigated Brain Stimulation system to estimate the intensity of the TMS‐evoked electric field, which was always adjusted to less than 90 V/m.^33^, ^34^ One study used stimulation intensities of 120% of RMT,^27^ and one study used SI 1 mV for both CS and TS at LICI.^29^

#### 3.2.3 The results of single‐pulse TMS and LICI paradigms

Canali et al. examined the natural frequency in event‐related spectral perturbation (ERSP) by applying single‐pulse TMS to the left premotor cortex.^33^, ^34^ The results showed that the BD group had significantly decreased beta/gamma band responses in the frontal area than the HC group. Farzan et al.^29^ calculated an area under the rectified curve between 50 and 150 ms in the LICI paradigm and found no significant difference between the BD and HC groups. One study reported the suppression of Mu rhythm by stimulating M1, which observed changes in EMG and EEG responses reflecting the corresponding muscle activity during exercise observation.^27^ Here, the Mu rhythm is an arch‐shaped 8–13 Hz waveform that reflects the mirror neuron system and is characterized by suppression by exercise and sensory stimulation.

### 3.3 Summary of risk of bias

Figure S1 provides a summary of the risk of bias in the included studies. In terms of participant selection, all articles mentioned both the age and sex of the subjects. When addressing confounding variables, seven out of the nine studies did not adjust for medication, suggesting a potential high risk of bias. Regarding the measurement of exposure, every study included patients with BD who were diagnosed with the DSM criteria. Since all studies included in this study compared the results of TMS neurophysiological indices in the BD and HC groups, the presence or absence of blinding of outcome assessment is irrelevant. Importantly, none of the studies included in this study had missing data that would have affected the study results. With regard to selective outcome reporting, we determined that the risk was moderately present in all studies, as it is difficult to completely rule out the possibility of selecting favorable outcomes from various analyses.

## 4 DISCUSSION

### 4.1 Summary of this study

This systematic review revealed a neurophysiological impairment in patients with BD when compared with HC. We found a consistent reduction in SICI in the BD group compared to the HC group. This finding suggests a GABA‐A receptor‐mediated dysfunction in BD. Additionally, impairments in LICI, IHI, and CSP were observed, indicating possible GABA‐B receptor‐mediated neurophysiological dysfunctions in patients with BD. These TMS paradigms provide insights into the altered cortical inhibitory mechanisms that may underlie the pathophysiology of BD. These results are keeping in line with previous studies demonstrating GABAergic dysfunction in terms of postmortem samples,^36^, ^37^, ^38^, ^39^, ^40^ ^1^H‐MRS,^41^, ^42^, ^43^ and blood and CSF samples,^44^, ^45^, ^46^ as well as the pharmacological mechanism of action of mood stabilizers^47^ and lithium.^48^ On the other hand, there was no significant difference in ICF between the BD and HC groups, suggesting that patients with BD may have intact glutamatergic NMDA receptor‐mediated function or that potential glutamatergic dysfunction with other modalities, as reported in previous studies,^49^, ^50^ may not be detected by the TMS‐EMG neurophysiology.

### 4.2 GABA‐A receptor‐mediated neurophysiology

This review demonstrated that GABA‐A receptor‐mediated function, as assessed by the SICI paradigm in M1, could be reduced in patients with BD compared to HC. Studies using SICI have consistently shown a reduction in cortical inhibition in the BD group compared to the HC group, regardless of medication status.^28^, ^31^, ^32^, ^35^ This reduction was observed in both the manic and euthymic phases of patients with BD, suggesting that the reduction in cortical inhibition of SICI may not be influenced by mood phase. Furthermore, comparisons of SICI between the manic phase and first‐episode mania in remission showed no significant group differences.^32^, ^35^ Collectively, since these studies did not include SICI findings in the depressed phase, no definitive conclusion can be drawn at this point that SICI shows consistent findings regardless of mood phase. Either way, it seems that patients with BD have a decreased cortical inhibition of SICI, but further studies are needed to scrutinize this SICI finding.

Genetic research highlights the importance of mutations in GABA‐A receptor subunits such as α4, α5, β1, β3, ρ1, ρ3, and GABRα6 in BD.^51^, ^52^ Furthermore, increased expression of GABRα1, GABRα6, GABRɛ, and polymorphisms in GABRα5 is also associated with BD pathology, highlighting the importance of these receptors in this disorder.^53^, ^54^, ^55^ These genetic alterations in patients with BD may represent pathological molecular bases that lead to GABA‐A receptor dysfunction. Regarding the pharmacological treatment for BD, an atypical antipsychotic, olanzapine, approved for BD, is noted to act on the GABA‐A receptor.^56^, ^57^ Basic neuroscience studies using rat brain sections have also shown that olanzapine administration increases GABA‐A receptor density in the prefrontal cortex.^58^ On the other hand, olanzapine administration has also been reported to act on GABA‐A receptors in the hippocampus and temporal lobe, downregulating these receptors and reducing their density.^59^ In addition, it has been reported that olanzapine antagonizes GABA‐A receptors, thereby increasing the activation of these neurons, and thus may have the ability to reverse sensorimotor gating dysfunction.^57^, ^58^ These findings also suggest that olanzapine may exert its mood‐stabilizing effects by acting on GABA‐A receptors. Taken together, these findings implicate that GABA‐A receptor‐related functions play a role in the pathophysiology of BD.

### 4.3 GABA‐B receptor‐mediated neurophysiology

Investigations into TCI in patients with BD have predominantly utilized the IHI paradigm to understand the interhemispheric communication via the corpus callosum.^30^, ^31^, ^32^ The IHI is classified into the SIHI and the LIHI according to the length of the ISIs.^60^, ^61^ The two SIHI studies included in this review showed consistent reductions,^31^, ^32^ while the LIHI study showed no significant difference between the manic phase of the BD group and the HC group.^32^ SIHI occurs over shorter intervals and is believed to involve direct GABAergic inhibitory pathways, reflecting rapid, immediate inhibitory processes between hemispheres. LIHI, on the other hand, involves longer intervals and is thought to result from a more complex interplay of excitatory and inhibitory signals, possibly including GABA‐B receptor‐mediated inhibition.^62^ Therefore, it is imperative to consider the potential role of the SIHI paradigm in the pathophysiology of BD and continue to explore its association with GABA‐related receptors. While only three TMS‐EMG studies have assessed CSP in BD, two of them have reported that CSP was shortened in patients with BD in comparison with HCs.^30^, ^31^ Given that the CSP paradigm is implicated in GABA‐B receptor‐mediated cortical inhibition,^63^ these findings suggest that GABA‐Bergic neurotransmission may be involved in the pathophysiology of BD. Furthermore, among the studies included in this review, four studies conducted the LICI paradigm,^28^, ^29^, ^32^, ^35^ two of which indicated increased cortical inhibition in the manic phase of the BD group compared to the HC group.^28^, ^35^

This discrepancy between the TCI and LICI paradigms may be due to differences in the projection sites in each TMS paradigm that could have impacted the outcomes. CSP involves applying TMS pulse to both left M1 and right M1 to evaluate the period of inhibition from the onset of the MEP to the recuperation of any muscle activity.^64^, ^65^ The CSP paradigm involves several spinal cord inhibitory mechanisms, while the IHI paradigm is thought to originate from synapses to interhemispheric excitatory pathways that project from motor‐related cortex to contralateral M1 via the corpus callosum and from local inhibitory circuits targeting ipsilateral M1,^61^, ^66^ with subcortical projections also contributing.^67^ The LICI paradigm involves delivering paired‐pulse TMS over the ipsilateral M1 area, at a longer interval to assess intracortical inhibitory mechanisms.^68^ This method contrasts with CSP, which is elicited by a single‐pulse TMS and involves measuring the duration of muscle silence following the pulse. While CSP evaluates the inhibitory effect following a single stimulus and is associated with GABA‐B receptor‐mediated inhibition, LICI assesses the cumulative inhibitory effect of two stimuli separated by a specific interval, providing insight into different aspects of cortical inhibitory function.

Furthermore, the two studies examining the LICI^28^, ^35^ included unmedicated manic patients with BD, while the rest of the studies investigating the CSP and IHI^31^, ^32^ did not control their medications. The studies in which the CSP was conducted^30^ did not provide information on participants' medication status. In addition, the studies conducting the CSP and IHI^31^, ^32^ included the manic and euthymic phases of BD, which may have led to differences in results compared to other studies due to the lack of uniformity in disease phases. In addition, one study assessed LICI using TMS‐EEG in the DLPFC for the BD group on medication (e.g., risperidone, quetiapine, and olanzapine) and showed no significant relationship between the LICI and doses of antipsychotics.^29^ This finding suggests that LICI is not influenced by antipsychotics in this population. Furthermore, the results were consistent with the TMS‐EMG findings of M1 in the BD group on medication.^32^ However, in the BD group not on medication, the LICI effect of TMS‐EMG at M1 was reduced, indicating that medication may rescue the reduced cortical inhibition in the BD group.

Lithium is the gold standard for the treatment of BD,^69^ and carbamazepine is an anticonvulsant medication frequently utilized for the treatment of manic states in BD.^70^ Chronic administration of lithium or carbamazepine in rats was suggested to up‐regulate GABA‐B receptors.^71^ In addition, quetiapine is one of the antipsychotics used for the treatment of BD as a mood stabilizer.^72^ CSP was found to be prolonged in patients with schizophrenia after treatment with quetiapine.^73^ On another note, clozapine has recently garnered attention as a promising treatment of treatment‐resistant BD.^74^ A TMS‐EMG study examined the effect of clozapine on CSP during the treatment of treatment‐resistant schizophrenia and demonstrated a prolongation of CSP following the treatment.^75^ Those findings suggest its potential involvement via GABA‐B receptors,^76^ supporting our results. Taken together, these pharmacological aspects also suggest that GABA‐B receptor‐mediated dysfunction may also be involved in the pathogenesis of BD.

### 4.4 Glutamatergic NMDA receptor‐mediated neurophysiology

In this review, only one ICF study using the TMS‐EMG method for patients with BD in the euthymic phase was included, but no significant difference was found in ICF compared to HC.^31^ However, a host of studies have reported abnormalities in the glutamatergic neurotransmitter mechanisms in BD through various research modalities. Postmortem brain studies have reported abnormalities in the expression of proteins related to AMPA, NMDA, and kainate receptors, as well as abnormal synaptic density in various brain regions, including the striatum,^77^, ^78^ hippocampus,^37^, ^79^, ^80^, ^81^ and frontal cortex.^82^, ^83^, ^84^ Moreover, genetic studies have also reported genetic abnormalities associated with these glutamate receptors.^85^, ^86^, ^87^, ^88^, ^89^, ^90^, ^91^ Furthermore, three meta‐analyses on MRS studies^92^, ^93^, ^94^ have all shown increased levels of Glu and Glu+ glutamine in the anterior cingulate cortex in patients with BD, compared to HCs. Positron emission tomography studies have shown decreased occupancy of metabotropic glutamate receptor 5 in the prefrontal cortex of patients with BD compared to HCs.^95^ On top of that, studies using peripheral blood plasma from patients with BD have shown decreased glutathione levels acting on NMDA receptors compared to HCs.^96^, ^97^ Indirect evidence from pharmacological studies has also shown the involvement of antipsychotics and mood stabilizers in the glutamatergic system. The results of the ICF paradigm studies included in this review were not consistent with the results from the other modalities mentioned above. However, the number of ICF studies in BD is still limited, and in particular, only one study was conducted in the euthymic phase of patients with BD.^31^ In addition, this study was conducted using the TMS‐EMG method, targeting M1 exclusively. Therefore, it is necessary to conduct future studies using TMS‐EEG for brain regions including the dorsolateral prefrontal cortex, which is more associated with the pathophysiology of BD. It is known that psychotropics such as lithium, mood stabilizers, antipsychotics, and anticonvulsants used to treat BD may attenuate glutamatergic function through multiple mechanisms,^47^ although the ^1^H‐MRS meta‐analysis noted no significant difference between the medicated and unmedicated groups.^94^ Neurophysiological findings are still few in number and need to be accumulated further in the future.

### 4.5 Limitation

Only a few TMS‐EEG studies have been conducted in patients with BD, and there is the only one TMS‐EEG study that used paired‐pulse TMS.^29^ Due to the overall small number of studies in this review, a systematic review and meta‐analysis based on the classification of their mood phases (depressive phase, euthymic phase, manic phase, and mixed state) of BD could not be conducted. In addition, most studies had small sample sizes, with fewer than 20 participants. In the TMS studies included in this review, the method of determining thresholds and setting stimulus intensity varied across the studies, which did not allow for adequate control of the parameters of the stimulus conditions. Furthermore, the presence or absence and content of medication varied among the studies, which could be a confounding factor for the TMS neurophysiological findings.

### 4.6 Future direction

This review has revealed cortical inhibitory dysfunction in BD from the perspective of TMS neurophysiology. Nevertheless, most of the included studies were mainly TMS‐EMG studies on M1 regions, and only one study employing the TMS‐EEG method was conducted using the LICI paradigm. Therefore, future studies should apply more various TMS‐EEG paradigms to patients with BD and investigate cortical regions other than M1. In particular, we need to better understand the pathophysiology of BD by elucidating glutamatergic and GABAergic functions in the dorsolateral prefrontal cortex, which are involved in mood disorders. Moreover, as mentioned in the limitations, since previous BD‐TMS neurophysiological studies have only had small sample sizes, future TMS neurophysiological studies should be conducted with larger samples, at least 20 or more subjects. Furthermore, since the brain functions of BD patients can change drastically depending on their mood phase and medications administered at the time, it is necessary to conduct experiments under controlled conditions in the future. It is also necessary to generate more generalizable results by standardizing the differences in stimulus parameters and methodologies among laboratories in TMS neurophysiology experiments.

## 5 CONCLUSION

The results of this review suggest that GABA‐A and GABA‐B receptor‐mediated dysfunctions may be implicated in the pathophysiology of BD. Given the aforementioned limitations of the included studies, further progress in TMS neurophysiological research including TMS‐EEG is highly expected to shed light on the pathophysiological basis of BD.

## AUTHOR CONTRIBUTIONS

Keita Taniguchi: Investigation, formal analysis, and writing – original draft. Naotsugu Kaneko: Investigation and writing – original draft. Masataka Wada: Investigation and writing – original draft. Sotaro Moriyama: Investigation and formal analysis. Shinichiro Nakajima: Supervision and writing – review and editing. Masaru Mimura: Writing – review and editing. Yoshihiro Noda: Conceptualization, writing – review and editing, project administration, and supervision.

## FUNDING INFORMATION

This research received no external funding.

## CONFLICT OF INTEREST STATEMENT

The authors declare no conflicts of interest associated with this manuscript.

## ETHICS STATEMENT

Approval of the research protocol by an Institutional Reviewer Board: N/A.

Informed Consent: N/A.

Registry and the Registration No. of the study/trial: N/A.

Animal Studies: N/A.

## Supporting information


Figure S1

## Data Availability

Data sharing not applicable to this article as no datasets were generated or analysed during the current study.
