# Supplementary material for: Neurophysiological profiles of patients with bipolar disorders as probed with transcranial magnetic stimulation: A systematic review
Source: Neuropsychopharmacol Rep. 2024 Jun 26;44(3):572–84. doi: 10.1002/npr2.12458 (PMC11544454; doi:10.1002/npr2.12458)
Supplement: Supplementary file 1 — Figure S1 [file NPR2-44-572-s001.docx]

**Supplementary Materials**

|  | Participant Selection | Confounding Variables | Measurement of Exposure | Blinding of Outcome Assessment | Incomplete Outcome Data | Selective Outcome Reporting |
| --- | --- | --- | --- | --- | --- | --- |
| Andrews et al., 2016 |  |  |  |  |  |  |
| Basavaraju et al., 2017 |  |  |  |  |  |  |
| Basavaraju et al., 2019 |  |  |  |  |  |  |
| Canali et al., 2015 |  |  |  |  |  |  |
| Canali et al., 2017 |  |  |  |  |  |  |
| Farzan et al., 2010 |  |  |  |  |  |  |
| Howells et al. 2022 |  |  |  |  |  |  |
| Levinson et al., 2007 |  |  |  |  |  |  |
| Ruiz-Veguilla et al., 2016 |  |  |  |  |  |  |

**Supplementary Figure 1.** The results of the risk of bias for each included study.

For the risk of bias assessment, all four categories (i.e., participant selection, measurement of exposure, blinding of outcome assessments, and incomplete outcome data) showed "low risk"; however, confounding variables were judged "high risk" because the effect of the medication could not be excluded in three studies, and selective outcome reporting was judged "unknown" because the experimental protocols were not available. In the Supplementary Figure 1, green cells indicate low risk, yellow cells indicate medium risk, and red cells indicate high risk.
